# Pioneering Comparative Proteomic and Enzymatic Profiling of Amazonian Scorpion Venoms Enables the Isolation of Their First α-Ktx, Metalloprotease, and Phospholipase A_2_

**DOI:** 10.3390/toxins17080411

**Published:** 2025-08-15

**Authors:** Karla C. F. Bordon, Gabrielle C. Santos, Jonas G. Martins, Gisele A. Wiezel, Fernanda G. Amorim, Thomas Crasset, Damien Redureau, Loïc Quinton, Rudi E. L. Procópio, Eliane C. Arantes

**Affiliations:** 1 Department of BioMolecular Sciences, School of Pharmaceutical Sciences of Ribeirão Preto, University of São Paulo (USP), Ribeirão Preto 14040-903, SP, Brazil; gabriellecristinasantos06@gmail.com (G.C.S.); gisele.wiezel@gmail.com (G.A.W.); 2 Graduate Program in Genetics, Conservation and Evolutionary Biology (PPG GCBEv), National Institute for Amazon Research (INPA), Manaus 69067-375, AM, Brazil; jonasgama83@gmail.com; 3 Laboratory of Mass Spectrometry, MolSys Research Unit, University of Liège, B-4000 Liège, Belgium; fernandagamorim@gmail.com (F.G.A.); thomas.crasset@uliege.be (T.C.); dredureau@uliege.be (D.R.); loic.quinton@uliege.be (L.Q.); 4 Graduate Program in Biotechnology and Natural Resources of Amazon, University of the State of Amazonas (UEA), Manaus 69050-020, AM, Brazil; rudiprocopio@gmail.com

**Keywords:** toxinology, metallopeptidase, neuroactive peptides, geographic variation, venomics, Amazon rainforest, mass spectrometry, enzyme, Chactidae, Buthidae

## Abstract

Scorpionism is a growing public health concern in Brazil, with the Amazon region presenting the highest mortality rates but remaining understudied, especially regarding local scorpion venoms composition. This study presents the first comprehensive biochemical characterization of venoms from three Amazonian species—*Tityus metuendus* (TmetuV), *Tityus silvestris* (TsilvV), and *Brotheas amazonicus* (BamazV)—using an integrated approach combining Multi-Enzymatic Limited Digestion (MELD)-based bottom-up proteomics, high-resolution LC-MS/MS, chromatography, zymography, and enzymatic assays. *Tityus serrulatus* venom was included as a reference. Significant biochemical differences were observed: TsilvV was rich in 20–30 kDa proteins and showed strong metalloprotease activity; BamazV exhibited high molecular weight proteins and potent phospholipase A_2_ (PLA_2_) activity but lacked proteolytic and fibrinogenolytic activities; TmetuV showed the highest hyaluronidase activity and abundance of α-KTx neurotoxins. Zymography revealed a conserved ~45 kDa hyaluronidase in all species. Three novel components were partially characterized: BamazPLA_2_ (Group III PLA_2_), Tmetu1 (37-residue α-KTx), and TsilvMP_A (a metalloprotease homologous to antarease). This is the first application of MELD-based proteomics to Amazonian scorpion venoms, revealing molecular diversity and functional divergence within *Tityus* and *Brotheas*, emphasizing the need for region-specific antivenoms. These findings provide a foundation for future pharmacological studies and the discovery of bioactive peptides with therapeutic potential.

## 1. Introduction

Scorpionism is a major public health concern in Brazil, where scorpions are responsible for the majority of accidents involving venomous terrestrial animals, often resulting in severe symptoms and death [1]. Over the past two decades, the incidence of scorpionism has increased dramatically in northern Brazil (by nearly 50%), where the highest case severity has been consistently reported since the early 21st century [2]. The North region harbors 52% of Brazil’s scorpion fauna, with over 70 known species [3], including 48 recorded in the state of Amazonas alone [2,4]. Despite this remarkable biodiversity, the Amazon region remains significantly underexplored in terms of the biochemical and toxicological properties of its scorpion venoms, limiting our understanding of their environmental roles and medical significance [2].

Recent advances in proteomic and transcriptomic technologies have revealed complex and species-specific venom compositions across scorpion lineages [5]. Nevertheless, in Brazil, research on scorpion venoms is still predominantly focused on *Tityus serrulatus*, the species most associated with severe envenoming and the primary target of antivenom production, while other medically relevant or ecologically significant species are underrepresented [6]. Among Amazonian scorpions, only *T. obscurus* has been extensively studied [7,8,9]. In contrast, species such as *T. metuendus* and *T. silvestris* remain poorly characterized at both the biochemical and molecular levels [10,11], while *Brotheas amazonicus* is normally considered non-medically relevant [12], but its venom contains components capable of paralyzing insects [13], contributing to arthropod predation [14], which must be relevant from an environmental perspective and highlights the pharmacological potential of its toxins [2]. These three Amazonian species were selected for comprehensive biochemical analysis in the present study. The well-characterized *T. serrulatus* venom was included as a comparative reference, allowing us to benchmark Amazonian venom profiles against a medically significant and thoroughly studied species.

Given the habitat diversity and evolutionary divergence among Amazonian scorpions, it is plausible that their venom profiles demonstrate adaptive traits distinct from those of *T. serrulatus*, with implications for both envenoming pathology and biotechnological exploitation.

Traditionally, scorpion venom research has focused on neurotoxins targeting voltage-gated ion channels, particularly those affecting sodium and potassium ion channels [15]. However, scorpion venoms are chemically complex secretions that also contain a broad array of peptides, small organic molecules (such as nucleosides and citric acid) [16], and enzymes involved in venom spreading, prey immobilization, and tissue disruption [17]. Among the enzymatic components, phospholipases, hyaluronidases, metalloproteases, and serine proteases play central roles in venom function and pathophysiology [17].

Studies on *T. serrulatus* and *T. metuendus* venoms have identified additional enzymatic components, including angiotensin-converting enzyme (ACE), endothelin-converting enzyme (ECE), carboxypeptidase, aminopeptidase A [18], cysteine and serine proteases (trypsin-like), lysozyme, chymotrypsin-like proteases, amylase, phosphodiesterase (PDE), chitinase, pancreatic lipase, phospholipases C (PLC) and D (PLD), amidating enzymes, and protease inhibitors [6]. These enzymes may participate in various biological processes, including protein degradation, membrane disruption, immunomodulation, and prey digestion.

Metalloproteases, in particular, stand out in *T. serrulatus* venom [5], as they cleave fibrinogen and neuropeptides, contributing to indirect neurotoxic and inflammatory effects [19,20]. One such metalloprotease, antarease, cleaves Soluble N-ethylmaleimide-sensitive factor Attachment Receptor (SNARE) proteins in exocrine tissues and has been implicated in post-envenoming pancreatitis [21]. Although metalloproteases are also present in *T. metuendus*, their biological roles remain uncharacterized [6,18].

Hyaluronidases, detected in both *T. serrulatus* and *T. metuendus* venoms [6], facilitate the diffusion of venom components through the extracellular matrix and may exhibit activity variations depending on environmental factors such as diet, as shown in *T. serrulatus* specimens fed with crickets versus cockroaches [22]. Additionally, phospholipase A_2_ (PLA_2_) has been identified in *T. serrulatus* venom through proteomic and transcriptomic approaches [6]. PLA_2_s may contribute to scorpion envenomation by inducing hemolytic, cytotoxic, and inflammatory effects, either through phospholipid hydrolysis or by triggering intracellular signaling pathways [23].

Despite these advances, a systematic comparative analysis of enzymatic activities among Amazonian scorpions remains lacking. To bridge this gap, the present study aimed to perform a comparative biochemical characterization of the enzymatic composition of *T. metuendus*, *T. silvestris*, and *B. amazonicus* venoms to the medically relevant *T. serrulatus*. We hypothesized that the venoms of Amazonian *Tityus* species exhibit distinct enzymatic profiles shaped by adaptive pressures and phylogenetic divergence. Moreover, given its distant taxonomic position and unique habitat niche, we expected that *B. amazonicus* venom would display a divergent enzymatic repertoire with specific biochemical features.

To test these hypotheses, we adopted a comprehensive and integrative analytical framework, combining reversed-phase fast protein liquid chromatography (FPLC), electrophoretic profiling (PAGE and SDS-PAGE), and targeted enzymatic assays. Notably, this study marks the first application of a MELD-based bottom-up proteomics workflow, coupled with high-resolution LC-MS/MS, to the analysis of Amazonian scorpion venoms. This multifaceted approach enabled the detailed identification and partial structural characterization of previously unreported venom components, allowing us to uncover distinct enzymatic signatures that reflect both functional adaptations and phylogenetic divergence. By expanding the biochemical understanding of these neglected species, our findings provide critical insights into venom composition, support improved toxicological assessment, and open promising avenues for bioprospecting. Ultimately, this work lays the foundation for the development of region-specific antivenoms and the discovery of novel enzymes and peptides with therapeutic or biotechnological potential.

## 2. Results

### 2.1. Chromatographic and Electrophoretic Profiles of Scorpion Venoms

**Figure 1:**
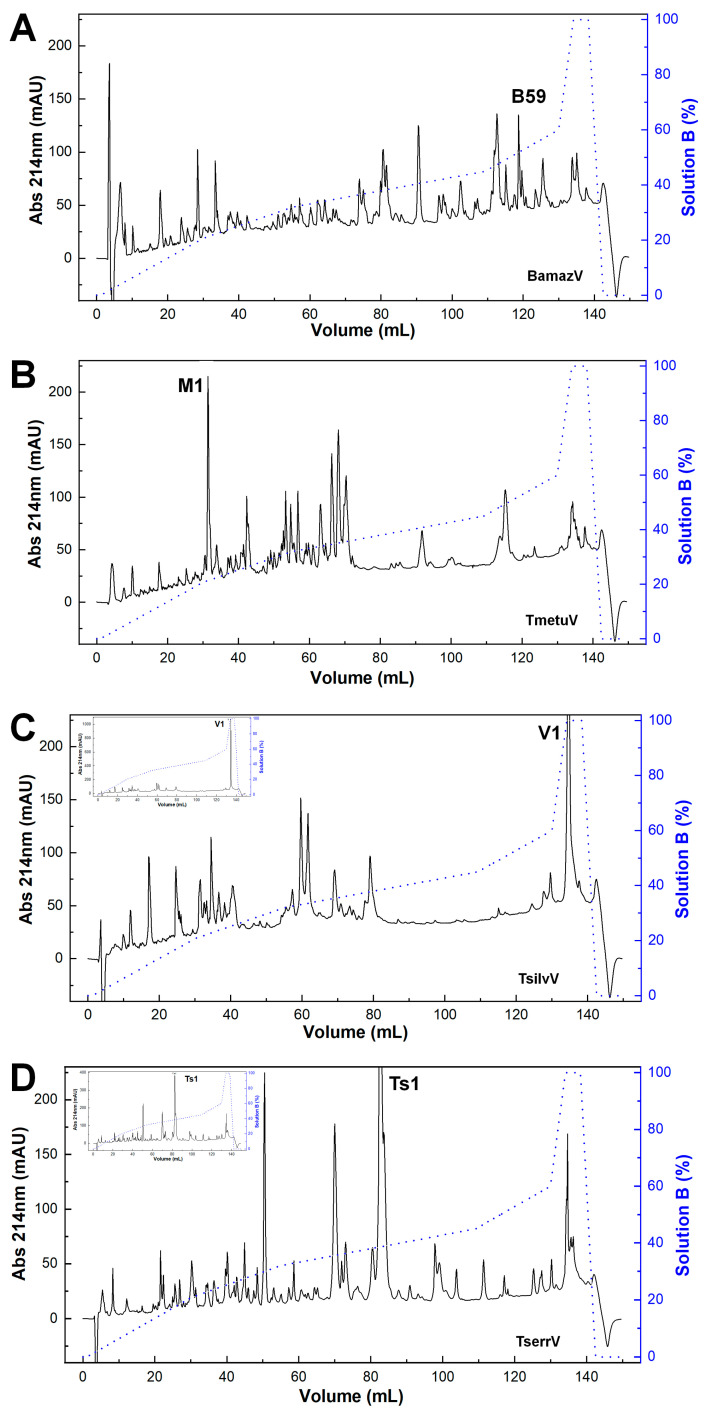
Chromatographic profiles of scorpion venoms. Crude soluble venoms (240 µg total protein) were fractionated on a C18 reversed-phase column (250 × 4.6 mm, 5 µm, 300 Å; Jupiter^®^, Phenomenex, Torrance, CA, USA) using an ÄKTA Basic FPLC system (GE Healthcare, Uppsala, Sweden). Elution employed a stepwise gradient (blue line) from 0 to 100% solution B (80% acetonitrile in 0.1% trifluoroacetic acid) at 1 mL/min. Absorbance was monitored at 214 nm. Chromatograms represent consistent replicate profiles. (**A**) BamazV; (**B**) TmetuV; (**C**) TsilvV; (**D**) TserrV. Insets in (**C**,**D**) show full absorbance traces, revealing maximum peaks of >1000 mAU (TsilvV) and ~400 mAU (TserrV). For comparison, y-axes in (**A**–**D**) were capped at 230 mAU; main traces in (**C**,**D**) thus represent scaled views.

The reversed-phase chromatographic profiles revealed pronounced interspecific variation in venom composition (Figure 1). BamazV (Figure 1A) displayed a markedly distinct elution profile compared to TserrV (Figure 1D), characterized by several prominent late-eluting peaks and a high recovery yield after 90 mL of elution. TmetuV (Figure 1B) was dominated by a single early-eluting peak (peak M1), representing 6.1% of the total protein recovery. No peaks were observed around 80 mL, corresponding to the elution volume of Ts1 in TserrV. TsilvV (Figure 1C) showed peak V1 as its dominant fraction, with a high protein recovery of 9.1%.

**Figure 2:**
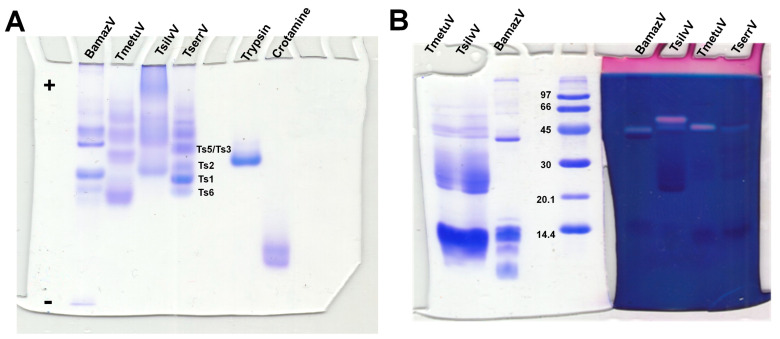
Electrophoretic and hyaluronidase activity profiling of scorpion venoms. (**A**) Native polyacrylamide gel electrophoresis (10%) under non-denaturing conditions optimized for basic proteins. Scorpion venoms (15 µg total protein): 1—BamazV, 2—TmetuV, 3—TsilvV, 4—TserrV. Controls: 5—trypsin (12 µg), 6—crotamine (8 µg). Running conditions: 100 V, 7.0–5.0 mA, 1 h 13 min. Proteins were visualized with 0.2% PlusOne Coomassie PhastGel™ Blue R-350. Bands corresponding to reference neurotoxins (Ts1, Ts2, Ts3/Ts5, Ts6) in TserrV are labeled. The differential migration patterns highlight the diversity in charge and molecular weight among venom components. (**B**) SDS-PAGE zymography showing hyaluronidase activity. Gels were co-polymerized with 0.4 mg/mL hyaluronic acid. Lanes 1 and 7—TmetuV; 2 and 6—TsilvV; 3 and 5—BamazV (20 and 40 µg total protein, respectively); 4—molecular weight marker (GE Healthcare, Uppsala, Sweden, 14–96 kDa); 8—TserrV (18 µg). Electrophoresis was performed at 110 V (14–6.7 mA) for 1 h. Gel portions were stained with either Coomassie (lanes 1–4) or Stains-All^®^ (lanes 5–8) to visualize protein content and hyaluronan-degrading activity, respectively.

Native PAGE under non-denaturing, low-pH conditions (Figure 2A) revealed substantial interspecific variation in venom composition and electrophoretic behavior. BamazV (lane 1) presented multiple distinct bands migrating toward the cathode, indicative of a predominance of basic proteins, consistent with low molecular weight proteins. TmetuV (lane 2) exhibited a broader and more diffuse banding pattern, suggesting compositional complexity and heterogeneity in charge. In contrast, TsilvV (lane 3) displayed bands migrating toward the anode, indicating the presence of more acidic proteins, likely corresponding to higher-molecular-weight components. TserrV (lane 4) served as a reference, showing discrete bands assigned to well-characterized neurotoxins Ts1, Ts2, Ts3/Ts5, and Ts6, validating the gel’s resolution and the preservation of native protein properties.

SDS-PAGE analysis (Figure 2B) further revealed differences in molecular mass distribution, particularly for BamazV, which showed a prominent band near 45 kDa and others below 14.4 kDa. However, due to the limited resolution of this 10% SDS-PAGE, it is not possible to clearly distinguish species-specific banding patterns for TmetuV and TsilvV. Therefore, the clearer interspecific differences in venom profiles were best supported by the native gel (Figure 2A).

### 2.2. Enzyme Activity

**Figure 3:**
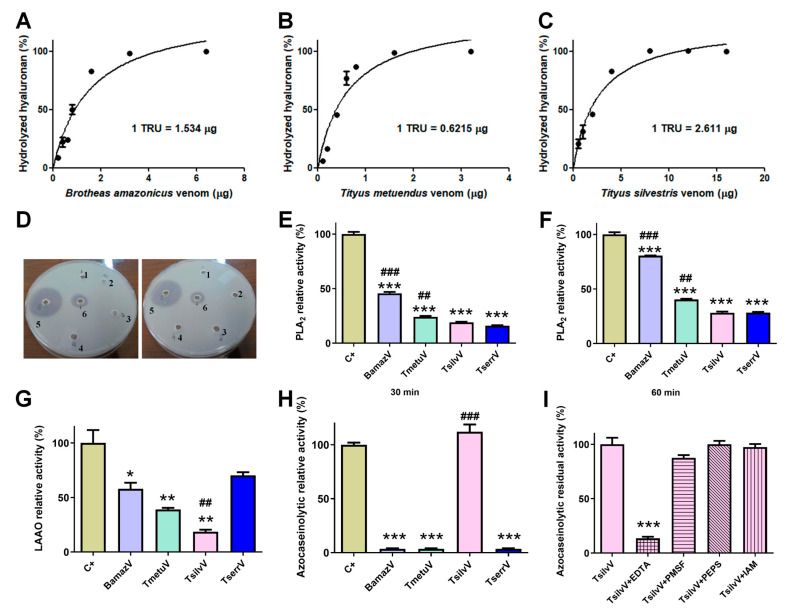
Enzymatic profiling of crude soluble venoms. (**A**–**C**) Quantitative turbidimetric hyaluronidase activity assays. Serial dilutions of (**A**) BamazV (0.2–6.4 µg/well), (**B**) TmetuV (0.1–3.2 µg/well), and (**C**) TsilvV (0.5–16.0 µg/well) were incubated in 200 mM sodium acetate buffer (pH 5.5) containing 200 mM NaCl and 0.5 mg/mL hyaluronan. Reactions were stopped with CTAB/NaOH, and absorbance was measured at 400 nm. One turbidity reducing unit (TRU) corresponds to the amount of enzyme required to hydrolyze 50% of hyaluronan. Specific activity was expressed as TRU/mg venom. (**D**–**F**) Comparative assessment of phospholipase A_2_ (PLA_2_) activity. (**D**) Qualitative egg yolk agar diffusion assay. 1—negative control, soluble venoms (65 µg/50 µL), 2—TserrV, 3—TsilvV, 4—TmetuV, 5—BamazV, and 6—positive control, were applied to agar plates containing egg yolk as phospholipid substrate. Plates were incubated at 37 °C for 16 h. Enzymatic activity was assessed by the formation of lysis halos, indicative of phospholipid hydrolysis. Assays were performed in duplicate. (**E**,**F**) Colorimetric assay using the synthetic substrate NOB. Venoms (50 µg/well) from BamazV, TmetuV, TsilvV, and TserrV were incubated with 5 mM NOB in 50 mM Tris-HCl buffer (pH 7.0) at 37 °C. Absorbance at 425 nm was measured after 30 min (**E**) and 60 min (**F**). Relative activity (%) was calculated in comparison to CdtV (set as 100%). (**G**–**I**) TsilvV was tested at a lower concentration (78 µg/well) due to limited venom availability. Nevertheless, activity was normalized to total protein content. (**G**) L-amino acid oxidase (LAAO) activity. Reactions containing venoms from BamazV, TmetuV, and TserrV (100 µg/well), and TsilvV (78 µg/well) in combination with 5 mM L-leucine in 50 mM Tris-HCl buffer (pH 7.0) were used to evaluate enzyme activity. LAAO activity was measured by H_2_O_2_ production using a peroxidase-coupled OPD oxidation system in 96-well microplates. Absorbance was read at 492 nm (ref. 630 nm). Reactions were stopped with 2 M H_2_SO_4_ after 60 min at 37 °C. (**H**,**I**) Proteolytic activity assessed by azocasein hydrolysis. (**H**) Relative proteolytic activity of scorpion venoms. BamazV, TmetuV, and TserrV (100 µg/well), and TsilvV (78 µg/well) were incubated with azocasein (425 µg/well) in 50 mM Tris-HCl buffer (pH 7.0) at 37 °C for 90 min. Absorbance was measured at 450 nm after precipitation with 5% TCA and color development with 0.5 M NaOH. Relative activity (%) was calculated in comparison to C+ (set as 100%). (**I**) Residual proteolytic activity of TsilvV in the presence of class-specific protease inhibitors. TsilvV was pre-incubated with 0.1 mM pepstatin A or 5 mM EDTA, PMSF, or iodoacetamide. Residual activity (%) represents the remaining enzymatic activity relative to uninhibited TsilvV (set as 100%). CdtV was used as the positive control (C+) in all enzymatic assays, except for the azocaseinolytic assay, in which trypsin served as the C+. Data are expressed as mean ± standard deviation (SD) of three independent experiments. Statistical significance was assessed by one-way ANOVA followed by Tukey’s post hoc test (* *p* < 0.05, ** *p* < 0.01, *** *p* < 0.001 vs. C+; ## *p* < 0.01, ### *p* < 0.001 vs. TserrV).

Zymography revealed hyaluronidase activity in BamazV, TmetuV, and TserrV, with a prominent band near 45 kDa (Figure 2B). TsilvV displayed two enzymatically active bands, one at around 45 kDa and another slightly higher. Turbidimetric assays (Figure 3A–C) confirmed interspecific variation: TsilvV (383 turbidity reducing units per milligram, TRU/mg), BamazV (652 TRU/mg), and TmetuV (1609 TRU/mg).

Phospholipase activity was evaluated via agar diffusion and colorimetric assays. In the egg yolk agar method, only BamazV induced a transparent lysis halo (well 5, Figure 3D), clearly visible on the opaque agarose matrix and significantly larger than that of the positive control (well 6), indicative of robust phospholipase A_2_-like activity. On the other hand, *Tityus* venoms showed no detectable activity under the same conditions. In contrast, colorimetric analysis using the synthetic substrate NOB revealed measurable PLA_2_ activity in all tested venoms (50 µg/well), with BamazV consistently displaying the highest enzymatic activity (Figure 3E,F). After 30 min of incubation, BamazV reached approximately 45% of the activity of the positive control (CdtV), and TmetuV and TsilvV showed intermediate activity, both significantly greater than that of TserrV (Figure 3E). After 60 min of incubation, all venoms exhibited increased PLA_2_ activity. Although these values were statistically different from the positive control, BamazV reached approximately 75% of the positive control’s activity (Figure 3F). A PLA_2_ component identified in Peak B59 of BamazV shows sequence similarity with HgPLA_2_ (Figure S1A; see Section 2.4.2), suggesting a conserved functional motif.

L-amino acid oxidase (LAAO) activity was detected using a colorimetric assay with the L-leucine substrate. TserrV exhibited the highest enzymatic activity, not statistically different from the positive control (around 65% of the positive control’s activity) (Figure 3G). In contrast, TsilvV showed only marginal activity (approximately 15%), which was significantly lower than that of TserrV (*p* < 0.05) (Figure 3G). No phosphodiesterase activity was detected in any venom, even at high concentrations.

### 2.3. Proteolytic Activity

Azocaseinolytic assays revealed distinct proteolytic profiles among the tested scorpion venoms. While TsilvV exhibited strong proteolytic activity, exceeding 100% relative to the positive control (trypsin), this difference was not statistically significant. No detectable activity was observed for BamazV, TmetuV, or TserrV under the same experimental conditions (Figure 3H). Functional classification using specific inhibitors showed that the azocaseinolytic activity of TsilvV was almost completely abrogated by ethylenediaminetetraacetic acid (EDTA), with high statistical significance compared to the positive control. A non-statistically significant partial inhibition was observed with phenylmethylsulfonyl fluoride (PMSF), while pepstatin A and iodoacetamide had no detectable effect on enzymatic activity (Figure 3I).

**Figure 4:**
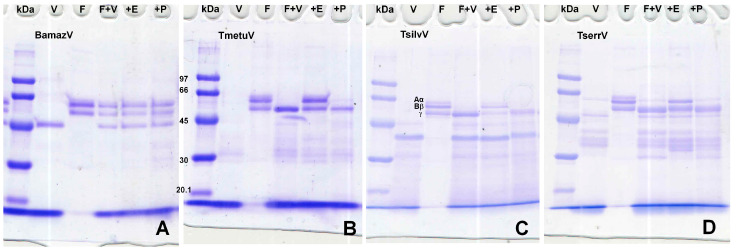
(**A**–**D**) Fibrinogenolytic activity assessed by SDS-PAGE (10%). V—venom alone (15 µg); F—fibrinogen alone (5 µg); F + V—fibrinogen (5 µg) + venom (15 µg); +E—fibrinogen (5 µg) + venom (15 µg) + 10 mM EDTA; +P—fibrinogen (5 µg) + venom (15 µg) + 10 mM PMSF. Reactions were carried out in 100 mM Tris-HCl buffer (pH 8.0) at 37 °C for 60 min and stopped with 3× Laemmli sample buffer containing β-mercaptoethanol, followed by heating at 100 °C for 5 min. Proteins were separated on a 10% SDS-PAGE gel under reducing conditions and stained with 0.2% PlusOne Coomassie PhastGel™ Blue R-350. Degradation patterns were analyzed by the disappearance or reduction in intensity of the fibrinogen chains. (**A**) BamazV; (**B**) TmetuV; (**C**) TsilvV; (**D**) TserrV.

Fibrinogen degradation assays (Figure 4) showed that BamazV induced no cleavage of α, β, or γ chains. *Tityus* species (TmetuV, TsilvV, and TserrV) showed selective cleavage of α and β chains with activity inhibition in the presence of EDTA.

### 2.4. Primary Structure and Molecular Identification of Major Venom Components

#### 2.4.1. α-KTx in TmetuV

Peak M1, the most abundant early-eluting fraction in TmetuV (hereafter referred to as Tmetu1), was identified as a 37-residue peptide by Edman degradation. Sequence alignment revealed high identity with TdK3, an α-KTx toxin from *Tityus discrepans*, and confirmed that Tmetu1 retains the conserved cysteine scaffold and predicted secondary structure elements (α-helix and β-strands) characteristic of the α-KTx family (Figure S1B), strongly suggesting it adopts a similar inhibitory fold. The protein sequence data of Tmetu1 reported in this paper will appear in the UniProt Knowledgebase under the accession number C0HMF6.

#### 2.4.2. PLA_2_ in BamazV

Peak B59, the most abundant late-eluting fraction of BamazV, contains a PLA_2_ (hereafter referred to as BamazPLA_2_) that shares 93.9% sequence similarity to HgPLA2 from *Hoffmannihadrurus gertschi* (formerly *Hadrurus gertschi*) venom, a representative member of the Group III heterodimeric PLA_2_ subfamily (Figure S1A). NetNGlyc 1.0 Server predicted a potential N-glycosylation site at position 12 (NEST motif) in the BamazPLA_2_ sequence, with a score of 0.6310 and 8/9 jury agreement. The protein sequence data of BamazPLA_2_ reported in this paper will appear in the UniProt Knowledgebase under the accession number C0HMF5.

#### 2.4.3. Metalloprotease in TsilvV

Peak V1 (hereafter referred to as TsilvMP_A), the most abundant late-eluting component of TsilvV, suggesting high hydrophobicity, displayed sequence similarity to antarease, a zinc metalloprotease from TserrV, and ~26 kDa homologs in other *Tityus* species (Figure S1C). The protein sequence data of TsilvMP_A reported in this paper will appear in the UniProt Knowledgebase under the accession number C0HMF7.

### 2.5. Relative Abundance of Toxin Classes

**Figure 5:**
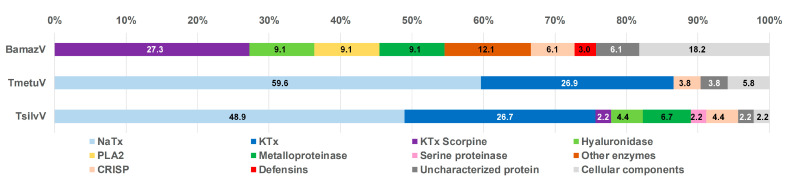
Functional classification of venom proteins from BamazV, TmetuV, and TsilvV based on mass spectrometry analysis. Protein families were categorized according to their known biological functions, including neurotoxins (NaTx, KTx, and KTx-Scorpine), enzymatic components (PLA_2_, metalloproteases, serine proteases, hyaluronidase, and other enzymes), defensins, and cellular components. The category “Uncharacterized proteins” includes sequences with no functional annotation. Percentages represent the relative abundance of each protein family in the total venom proteome.

Based on mass spectrometry analysis, the relative abundance of each toxin class was inferred from the proportion of assigned spectra (Figure 5). While not equivalent to absolute protein quantification, this approach enables comparative assessment of the major protein classes present in the tested venoms, revealing marked interspecific differences in venom composition.

TmetuV and TsilvV were predominantly neurotoxic, comprising large proportions of NaTx (above 48%) and KTx peptides (above 26%), consistent with their known clinical severity. In contrast, BamazV lacked detectable NaTx and classical KTx peptides, containing only KTx scorpine-like peptides (around 27%), which are functionally distinct toxins with antimicrobial properties and modest potassium channel blockade.

BamazV displayed a venom profile enriched in enzymatic components, notably metalloproteases, PLA_2_, and hyaluronidase (9.1% of each), suggesting a mode of action centered on tissue degradation and prey digestion. Furthermore, BamazV exhibited the highest proportions of uncharacterized proteins (6.1%) and cellular components (18.2%). These included proteins such as fatty acid-binding protein, profilin, and peptidyl-prolyl cis-trans isomerase, which, although often considered housekeeping molecules, are increasingly recognized as genuine venom components with potential roles in lipid transport, cytoskeletal modulation, and protein folding during venom biosynthesis or secretion.

The Venn diagram (Figure S2) revealed distinct patterns of protein distribution among *T. silvestris, T. metuendus*, and *B. amazonicus*. *T. metuendus* presented the highest number of unique proteins (85), while *T. silvestris* and *B. amazonicus* each exhibited 32 exclusive proteins. A notable overlap of 18 proteins was observed between *T. silvestris* and *T. metuendus*, whereas *B. amazonicus* shared only one protein with both *Tityus* species and no proteins exclusively with either. Comparative lists of peptides and proteins identified in each Amazonian scorpion venom are available in the Supplementary Material (File S2, comprising four .xlsx files).

## 3. Discussion

Biochemical profiling of *Tityus* and *Brotheas* venoms revealed pronounced interspecific divergence in toxin composition and enzymatic strategies, signifying distinct molecular specializations. Integrated chromatographic and electrophoretic analyses uncovered unique signatures in protein hydrophobicity, molecular weight, and charge distribution across species.

TmetuV exhibited a dominant early-eluting chromatographic peak (M1), an abundance of low molecular weight components (6.5–14.4 kDa), and a native PAGE profile enriched in basic peptides. These features are consistent with a neurotoxic venom composition dominated by α-KTx peptides. The main component, Tmetu1, is a 37-residue α-KTx peptide sharing 72% identity with TdK3 from *T. discrepans* and high similarity to Tc32 from *T. obscurus*, a potent Kv1.3 blocker (Kd = 10 nM) in human T lymphocytes [24]. While Tc32 is highly active, TdK3 exhibits low potency and selectivity toward Shaker B and Kv1.3 channels [25], suggesting that Tmetu1 may have unique pharmacological properties. The conserved structure of Tmetu1 underscores the evolutionary stability of the α-KTxs scaffold and its neuropharmacological relevance [26,27].

In contrast, TsilvV displayed electrophoretic and chromatographic profiles enriched in acidic, higher-molecular-weight components. Native PAGE revealed protein migration toward the anode, and SDS-PAGE showed a dominant protein population in the 20–30 kDa range, consistent with the molecular profile of metalloproteases. Chromatography showed late-eluting, hydrophobic fractions, notably peak V1. Edman degradation of V1 revealed high similarity to known metalloproteases. It was designated as TsilvMP_A, a metalloprotease homologous to ~26 kDa antarease-like toxins (Figure S1C), implicated in fibrinogenolysis [28], extracellular matrix remodeling [19], and neurotransmitter modulation [29]. Functional assays confirmed EDTA-sensitive proteolytic activity, supporting its classification as a zinc-dependent metalloprotease. Additional findings included two active hyaluronidase isoforms (Figure 2B) and absence of significant LAAO activity, indicating a venom modus operandi focused on tissue degradation and diffusion, possibly associated with microhabitat adaptations. From a translational standpoint, TsilvMP_A may serve as a candidate for therapeutic exploration in tissue remodeling and protease inhibition, pending further studies on physiological substrates such as collagen or elastin.

Similarly, BamazV displayed a highly complex chromatographic profile with multiple late-eluting peaks indicative of high-molecular-weight and/or hydrophobic components. Peak B59 (Figure 1A) was identified as a Group III PLA_2_ (BamazPLA_2_) sharing high sequence similarity with HgPLA_2_ from *Hoffmannihadrurus gertschi* (Figure S1A). Group III PLA_2_s are heterodimeric enzymes unique to arachnids and structurally distinct from monomeric PLA_2_s in snake venoms. It consists of a long catalytic chain linked by a disulfide bond to a shorter chain formed after proteolytic removal of a pentapeptide during post-translational maturation [23], differing from Group IA and IIA enzymes in elapids and viperids. Mass spectrometry of Phaiodactylipin, a Group III PLA_2_ from the scorpion *Anuroctonus phaiodactylus*, revealed N-linked glycosylation with three hexoses, two N-acetylhexosamines, and two deoxyhexoses [30]. Consistently, BamazPLA_2_ features a predicted N-glycosylation site at position 12 (NEST motif, Figure S1A), and the discrepancy between the theoretical (11.3–11.6 kDa) and experimental MALDI-TOF masses (14–19 kDa) for homologous sequences (Figure S1A) supports conserved glycosylation as a structural hallmark. Despite limited characterization of Group III PLA_2_s, arachnid enzymes exhibit diverse activities, including neurotoxic, myotoxic, hemolytic, anticoagulant, anti-angiogenic, and antitumor effects [23,31].

Functional assays confirmed strong PLA_2_ activity in BamazV, evidenced by a transparent lysis halo on the opaque egg yolk agar matrix (well 5, Figure 3D) and significant hydrolysis in colorimetric assays using NOB (Figure 3E,F), achieving ~75% of *Crotalus durissus terrificus* venom activity at 60 min. The egg yolk assay cannot distinguish between PLA_2_, PLC, or PLD activity [32], but the presence of Group III PLA_2_s identified by mass spectrometry (Supplementary Materials, File S2) strongly supports its role as the predominant active component. BamazPLA_2_ shares 61% identity and 85% similarity with IpTx1, a well-characterized from *Opisthacanthus cayaporum*, a Group III sPLA_2_ known to inhibit ryanodine receptors (RyR), induce hemolysis, and exert anticoagulant effects [30,33], reinforcing its functional and pharmacological relevance.

In contrast, TmetuV, TsilvV, and TserrV exhibited lower PLA_2_ activity and slower kinetics. These findings highlight interspecific variation in enzymatic strategies, with BamazV showing a proteolytic profile potentially shaped by distinct natural system demands. TserrV exhibited the highest LAAO activity (Figure 3G), moderate hyaluronidase and PLA_2_ activities, confirming its multifunctional toxin repertoire. and a dominant component (Ts1) accounting for 16% of total protein [22]. Notably, Ts1 (~6.8 kDa, pI 8.67), which elutes at around 80 mL, and related toxins were absent in BamazV, TmetuV, and TsilvV, reinforcing their biochemical divergence.

BamazV also exhibited moderate LAAO activity (Figure 3G), aligning with previous reports in arachnid venoms [34,35]. LAAO activity may contribute to oxidative cytotoxicity and immunomodulation. The presence of these enzymes, especially in TserrV and BamazV, highlights venom multifunctionality and their potential biomedical applications.

Notably, only TsilvV exhibited detectable activity against azocasein, a broad-spectrum protease substrate. This activity was strongly inhibited by EDTA and partially by PMSF, indicating dominant zinc-metalloprotease activity with minor serine protease contribution. The ineffectiveness of pepstatin A and iodoacetamide suggests minimal contributions from aspartic and cysteine proteases. TmetuV, TserrV, and BamazV lacked detectable azocaseinolytic activity. However, all *Tityus* venoms cleaved fibrinogen chains in SDS-PAGE analysis (Figure 4), with activity inhibited by EDTA, confirming the presence of functionally active metalloproteases capable of targeting coagulation factors.

Several methodological and biological inconsistencies warrant discussion. First, the proteomic detection of metalloproteases in BamazV (9.1% spectral abundance, Figure 5) conflicted with their absence of activity in functional assays, suggesting limitations in correlating MS data with enzymatic activity (e.g., zymogen states or unoptimized assay conditions). Second, the marked variation in PLA_2_ activity among venoms may reflect differential enzyme expression, natural inhibitors, or uncharacterized post-translational modifications. Third, while PDEs were identified in TserrV (0.6% of soluble venom) [6], no phosphodiesterase activity was detected, a discrepancy that may reflect their typically low abundance (<0.1–3.6% in snake venoms [36]) or assay sensitivity limitations. Finally, substrate-specific disparities (e.g., TsilvV’s azocaseinolytic activity vs. fibrinogenolysis in other venoms) underscore that functional profiles are context-dependent. These limitations highlight the need for complementary approaches, including targeted substrate screens and activation studies, to fully resolve venom bioactivity.

Notwithstanding these technical considerations, the biochemical divergence across species reveals distinct evolutionary trajectories. The dominance of α-KTxs in TmetuV, metalloproteases in TsilvV, and PLA_2_s in BamazV contrasts sharply with the NaTx-rich profile of TserrV, suggesting niche-specific adaptations shaped by ecological pressures. Although structurally related to α-KTxs, scorpine-like peptides identified in BamazV are functionally distinct, exhibiting dual antimicrobial and weak, non-selective K^+^ channel modulatory activity.

These differences carry translational implications, particularly regarding antivenom efficacy, as current therapies optimized for TserrV may poorly neutralize Amazonian species [37]. The identification of diverse cellular components and ancillary enzymes highlights the functional diversification of these venoms beyond classical neurotoxins. Notably, we detected NPC2-like protein in TsilvV, chitinase, hemocyanin, and fatty acid-binding protein in TmetuV, along with elevated proportions (~18%) of profilin, peptidyl-prolyl cis-trans isomerase, and additional fatty acid-binding proteins in BamazV. While often considered contaminants, these components likely serve biological roles in venom stabilization, host response modulation, and solubility. Furthermore, BamazV contains a remarkable array of digestive and immunomodulatory enzymes including α-amylase, α-L-fucosidase, histidine phosphatase, and angiotensinogenase, revealing a truly multifaceted venom arsenal with potential roles in both prey digestion and host manipulation.

Enzymatic profiling revealed conserved hyaluronidase activity across all species (~45 kDa), with notable interspecific variations in expression patterns and efficiency. While TsilvV showed two distinct active bands (Figure 2B), suggesting possible isoforms or post-translational modifications, quantitative assays showed TmetuV exhibited the highest specific activity (1609 TRU/mg), nearly double that of TserrV (845 TRU/mg) and significantly surpassing that of *Crotalus durissus terrificus* venom (145 TRU/mg) [38]. These substantial differences in hyaluronidase activity likely reflect adaptations for enhanced tissue penetration efficiency in their respective ecological niches.

Proteomic comparisons reveal significant overlap between *T. silvestris* and *T. metuendus* (Figure S2), suggesting shared ancestry, while *B. amazonicus* exhibits minimal overlap with *Tityus* species, underscoring its phylogenetic divergence.

Altogether, these findings emphasize the need to expand translational, pharmacological, and immunological studies beyond *T. serrulatus*. Such efforts will be essential for improving antivenom design and uncovering novel therapeutic tools from the rich biochemical diversity of Amazonian scorpion venoms.

## 4. Conclusions and Perspectives

This pioneering comparative analysis reveals a striking functional divergence among Amazonian scorpion venoms, shaped by evolutionary factors such as prey specialization, habitat complexity, and foraging strategies. TmetuV, enriched in α-KTx neurotoxins such as Tmetu1, targets potassium channels and exemplifies a mechanism focused on rapid neurotoxic immobilization. In contrast, TsilvV exhibits high proteolytic activity, led by the antarease-like metalloprotease TsilvMP_A and hyaluronidases, supporting tissue degradation and enhanced toxin diffusion—traits potentially advantageous in navigating dense forest environments or preying on arthropods with thick exoskeletons.

BamazV lacks canonical NaTx and α-KTx neurotoxins, and instead, it is dominated by a catalytically active Group III PLA_2_ (BamazPLA_2_) and scorpine-like peptides, suggesting an alternative adaptive strategy potentially shaped by distinct predator–prey dynamics in its arboreal microhabitat. This enzymatic dominance, coupled with its low clinical impact, reinforces how ecological niche specialization drives venom evolution.

Enzymatic activity profiling further illustrates this functional heterogeneity: hyaluronidases in TmetuV, metalloproteases in TsilvV, PLA_2_s in BamazV, and LAAOs in TserrV. Interestingly, activity did not always correlate with protein abundance, highlighting the importance of functional dominance over sheer proteomic presence. The detection of cellular proteins such as profilin, fatty acid-binding proteins, and peptidyl-prolyl isomerases, often considered as contaminants, suggests auxiliary roles in venom function, potentially contributing to structural stability, lipid transport, or immunomodulation.

Functionally, TsilvV exhibited the broadest proteolytic activity, including azocaseinolytic and fibrinogenolytic effects, supporting a role in extracellular matrix disruption. TmetuV displayed the highest hyaluronidase activity (1609 TRU/mg), facilitating venom diffusion through host tissues. These enzymatic signatures not only define venom function but provide measurable indicators of ecological adaptation pressures.

From a translational perspective, this study expands the known toxinological repertoire of Amazonian scorpions and introduces novel bioactive molecules. The isolation and biochemical characterization of Tmetu1, TsilvMP_A, and BamazPLA_2_ highlight their pharmaceutical promise, particularly TsilvMP_A for antifibrotic therapies and BamazPLA_2_ for immunomodulation applications.

To address the disconnection between proteomic detection and biological function—exemplified by the presence of metalloproteases in BamazV with no detectable enzymatic function—future studies should integrate multi-omics approaches with functional assays. This includes using fluorogenic or chromogenic substrates and zymography under native conditions, with priority given to activity-based protein profiling (ABPP; a chemical proteomics method to monitor enzyme activities in complex mixtures) to resolve zymogen activation mechanisms.

Importantly, the divergence between Amazonian *Tityus* species and the medically significant *T. serrulatus* raises concerns about current antivenom coverage. For clinical translation, immunization protocols incorporating TsilvMP_A and BamazPLA_2_ should be explored to enhance protection against Amazonian species, particularly given the low recognition of non-NaTx components in *T. metuendus* and *T. silvestris*.

Three critical avenues emerge for future research: (1) field studies characterizing ecological drivers of venom specialization across microhabitats, (2) systematic evaluation of antivenom cross-reactivity against Amazonian species, and (3) clinical development of TsilvMP_A and BamazPLA_2_ through structure-activity relationship studies. These priorities necessitate integrated approaches combining ABPP, targeted substrate screens, and the MELD-based proteomics validated here.

The MELD-based proteomic workflow proved effective in resolving the molecular complexity of these venoms. When integrated with ecological field data and in vivo assays, it will facilitate deeper insights into toxin evolution and therapeutic potential.

In conclusion, this study establishes both a biochemical foundation and a new research paradigm for understanding Amazonian scorpion venoms. These findings not only bridge fundamental toxinology and biomedical innovation but provide a template for studying neglected venomous taxa through multidisciplinary approaches, from molecular evolution to clinical translation.

## 5. Materials and Methods

### 5.1. Scorpion Venom Milking

Scorpion handling and venom collection complied with institutional guidelines, Brazilian legislation, and international standards for arthropod care. Adult specimens of *Tityus metuendus*, *T. silvestris*, and *Brotheas amazonicus* were collected in Manaus, Amazonas, Brazil (03°04′34″ S; 59°57′30″ W) and identified morphologically [39] under the Brazilian Biodiversity Information and Authorization System (SISBIO) permit No. 56748-1. *T. serrulatus* venom was obtained from the vivarium of the Ribeirão Preto campus of the University of São Paulo, under Brazilian Institute of Environment (IBAMA) registration No. 1506748. Venoms were extracted via mild electrical stimulation (12 V), lyophilized, and stored at −20 °C until use. This research complies with the Nagoya Protocol and Brazilian genetic heritage regulations, registered in the National System for Management of Genetic Heritage and Associated Traditional Knowledge (SISGEN, Ministry of the Environment) under No. A4A9FDD, ensuring ethical and legal access and use of biodiversity.

### 5.2. Preparation of Soluble Venoms

Crude lyophilized venoms from *Brotheas amazonicus* (BamazV), *Tityus metuendus* (TmetuV), *T. silvestris* (TsilvV), and *T. serrulatus* (TserrV) were weighed with precision and dispersed in ultrapure water (18.2 MΩ·cm, Milli-Q, Millipore, Burlington, MA, USA) to obtain the same theoretical concentrations. After centrifugation under the same conditions at 13,000× *g* for 5 min at 4 °C, variable amounts of insoluble material were observed across venom samples, indicating differences in solubility. The resulting supernatants, hereafter referred to as “crude soluble venoms”, were immediately transferred to sterile microtubes and kept on ice until use. Protein concentration was estimated by UV spectrophotometry using a NanoDrop 2000 system (Thermo Scientific, Waltham, MA, USA), based on the absorbance of the soluble fraction at 280 nm and a fixed specific extinction coefficient (ε₍_280_₎ = 1.0 mg^−1^·mL·cm^−1^). Due to the limited amount of venom, colorimetric assays such as Bradford or BCA could not be applied.

### 5.3. Purification of Crude Soluble Venoms Through Reversed-Phase Chromatography

Crude soluble venoms (240 µg of total proteins) were fractionated on a reversed-phase (RP) C18 column (250 × 4.6 mm, 5 µm particle size, 300 Å pore size; Jupiter^®^, Phenomenex, Torrance, CA, USA), connected to an ÄKTA Basic fast protein liquid chromatography (FPLC) system (GE Healthcare, Uppsala, Sweden). Elution was carried out at a flow rate of 1 mL/min using a stepwise concentration gradient from 0 to 100% of solution B (80% acetonitrile [MeCN] in 0.1% trifluoroacetic acid [TFA]), as previously reported [22]. Eluate absorbance was continuously monitored at 214 nm. Collected fractions were immediately frozen at −80 °C, lyophilized, and stored at −20 °C until use. RP-FPLC fractionation was independently repeated multiple times for each species, consistently yielding highly similar chromatographic profiles, thus demonstrating the reproducibility and reliability of the separation procedure.

### 5.4. Polyacrylamide Gel Electrophoresis (PAGE)

Native PAGE was employed to separate venom proteins based on their net charge under non-denaturing, low-pH conditions optimized for basic components. Polyacrylamide gels (10%, *w*/*V*) were prepared with an internal buffer of 50 mM potassium acetate (pH 4.5). Crude soluble venoms were diluted to a final 20% (*w*/*V*) sucrose and loaded with no reducing agents or denaturants. Electrophoresis was conducted in a discontinuous buffer system composed of 35 mM β–alanine–acetic acid (electrode buffer) and 50 mM potassium acetate (gel buffer), as originally described by Reisfeld et al. and later adapted for venom analysis [40]. A pre-electrophoresis stabilization step at 100 V for 50 min at room temperature was used to optimize resolution and minimize background noise. The analytical run was then continued under the same conditions until the basic fuchsin tracking dye reached the gel midpoint. This native PAGE system preserves protein conformations and provides enhanced resolution of basic, low-molecular-weight venom components. Gels were stained with 0.2% (*w*/*V*) PlusOne Coomassie PhastGel™ Blue R-350 (GE Healthcare, Uppsala, Sweden) for protein visualization.

### 5.5. Enzyme Activity Assays

#### 5.5.1. Hyaluronidase Activity Assays

##### Zymography Analysis

The soluble venoms (20 and 40 µg of total protein/well from each Amazonian species and 18 µg of TserrV venom, as a positive control) were loaded on a 10% SDS-PAGE co-polymerized with 0.4 mg/mL hyaluronic acid, following established methodologies with modifications [41,42]. Electrophoresis was carried out under non-reducing conditions at a constant voltage of 110 V at room temperature. A molecular weight marker (GE Healthcare, Uppsala, Sweden, 17-0446-01; 14–96 kDa) was included for molecular mass estimation. Following separation, part of the gel was stained with 0.025% Stains-All^®^ (Sigma Chemical Co., St. Louis, MO, USA) to reveal zones of hyaluronic acid degradation as clear bands on a violet background, and the remaining part with 0.2% PlusOne Coomassie Blue PhastGel™ R-350 (GE Healthcare, Uppsala, Sweden) for total protein profiling.

##### Turbidimetric Activity Assay

Quantitative hyaluronidase activity was assessed using a microplate-adapted turbidimetric assay, originally described by Diferrante and subsequently optimized for venom analysis [38,43,44]. Serial dilutions of soluble venom proteins (BamazV, 0.2–6.4 µg/well; TmetuV, 0.1–3.2 µg/well; and TsilvV, 0.5–16.0 µg/well) were incubated with 20 µL of 0.5 mg/mL hyaluronan in a final volume of 200 µL in 200 mM sodium acetate buffer (pH 6.0) containing 200 mM NaCl at 37 °C for 15 min. The reaction was stopped by adding 200 µL of 2.5% (*w*/*V*) cetyltrimethylammonium bromide (CTAB) in 2% (*w*/*V*) NaOH, which precipitates undigested hyaluronic acid, allowing turbidity measurements. Absorbance was measured at 400 nm using a microplate reader (Sunrise™, Tecan, Männedorf, Switzerland) within 10 min to avoid artifacts from delayed CTAB precipitation. All assays were performed in triplicate. One Turbidity Reducing Unit (TRU) was defined as the amount of venom required to hydrolyze 50% of hyaluronan. Specific activity was calculated as turbidity reducing units per milligram of total venom protein (TRU/mg) and determined using GraphPad Prism software, version 6.0 (GraphPad Software Inc., San Diego, CA, USA).

#### 5.5.2. Phospholipase A_2_ (PLA_2_) Activity Assays

##### Qualitative Egg Yolk Agar Plate Assay

PLA_2_ activity was qualitatively evaluated using an agar diffusion assay containing egg yolk as substrate [32]. Agar plates (90 × 15 mm Petri dishes) were prepared by pouring a warm mixture of 1.5% agar and 5% (*V*/*V*) hen egg yolk emulsion into the dish and allowing it to solidify at room temperature. Cylindrical wells were created in the gel using a 10 µL pipette tip. Each well was filled with 50 µL of scorpion venom solution containing 65 µg of total protein from BamazV, TmetuV, TsilvV, and TserrV. *Crotalus durissus terrificus* venom (CdtV, 10 µg total protein) was included as a positive control due to its well-characterized PLA_2_ activity, while ultrapure water served as a negative control. Plates were incubated at 37 °C for 16 h. Enzymatic activity was assessed by the formation of transparent lysis halos around the wells on the opaque egg yolk agar matrix, indicating phospholipid hydrolysis. Although the assay is qualitative, halo diameters provide a semi-quantitative estimate of relative enzymatic activity. All experiments were performed in duplicate to ensure reproducibility.

##### Colorimetric NOB Substrate Assay

PLA_2_ activity was also assessed using a colorimetric assay based on the hydrolysis of the synthetic substrate 4-nitro-3-octanoyloxybenzoic acid (NOB, 5 mM) following previously established procedures with adaptations for 96-well microplate format [45]. Reactions were performed in a final volume of 200 µL in 50 mM Tris-HCl buffer (pH 7.0). Crude soluble venoms from BamazV, TmetuV, and TserrV were tested at 50 µg/well. CdtV (5 µg/well) was used as a positive control, given its well-established PLA_2_ activity, while wells containing only buffer and substrate served as negative controls. The reaction mixtures were incubated at 37 °C, and absorbance at 425 nm was measured at 30 and 60 min using a microplate reader. All assays were conducted in triplicate, and background absorbance was subtracted from each well. PLA_2_ activity was expressed as relative activity (%), calculated based on the absorbance at 425 nm, with the activity of the positive control (CdtV) set as 100%.

#### 5.5.3. Proteolytic Activity Assays

##### Azocaseinolytic Activity

Proteolytic activity of scorpion venoms was evaluated using azocasein (425 µg/well) as a chromogenic substrate, adapted from established methodology [46] for use in 96-well microplates. Reactions were performed in a final volume of 200 µL in 50 mM Tris-HCl buffer (pH 7.0). Crude soluble venoms from BamazV, TmetuV, and TserrV were tested at 100 µg/well, whereas TsilvV was evaluated at a lower amount (78 µg/well) due to limited venom availability. Despite the reduced quantity, enzymatic activity was normalized to total protein content, and TsilvV still exhibited the highest activity among the samples. Trypsin (0.05 µg/well) was used as a positive control, and bovine serum albumin (BSA, 100 µg/well) served as a negative control, confirming assay specificity. To investigate the enzymatic class of the proteases involved, venoms were incubated in the absence or presence of protease class-selective inhibitors, each at final concentrations pre-established in the literature: 5 mM ethylenediaminetetraacetic acid (EDTA) for metalloproteases, 5 mM phenylmethylsulfonyl fluoride (PMSF) for serine proteases, 0.1 mM pepstatin A for aspartic proteases, and 5 mM iodoacetamide for cysteine proteases. Inhibitor preincubation was performed at 37 °C for 5 min before substrate addition. Reactions were initiated by the addition of azocasein and incubated for 90 min at 37 °C. To stop enzymatic activity and precipitate undigested substrate, 5% (*w*/*V*) trichloroacetic acid (TCA) was added. Samples were centrifuged at 1000× *g* for 5 min, and 100 µL of each supernatant was transferred to a microplate. Then, 100 µL of 0.5 M NaOH was added to develop color. Absorbance was measured at 450 nm using a microplate reader. All reactions were performed in technical triplicate, and background absorbance from blanks (buffer + substrate, no venom) was subtracted from all values before analysis.

Residual activity refers to the percentage of enzymatic activity that remains after treatment with specific inhibitors and is used to infer the protease class involved. It was calculated relative to the uninhibited venom control (set as 100%) using the formula: Residual activity (%) = 100 × (A_450_ inhibitor-treated/A_450_ untreated control). Relative activity, on the other hand, compares the proteolytic potential among different venom samples. In this study, it was calculated as the percentage of absorbance relative to the positive control (trypsin), which was defined as 100% proteolytic activity. Thus, relative activity (%) = 100 × (A_450_ sample/A_450_ trypsin). This approach allows for both intra- and inter-sample comparisons under standardized conditions and improves interpretability across experimental replicates.

##### Fibrinogenolytic Activity

Fibrinogenolytic activity of scorpion venoms was assessed by evaluating their ability to cleave the α, β, and γ chains of human fibrinogen using SDS-PAGE, following established protocols [47], with modifications. Briefly, human fibrinogen (5 µg/well) was incubated with crude soluble venoms (15 µg/well) from BamazV, TmetuV, TsilvV, and TserrV in 100 mM Tris-HCl buffer (pH 8.0) at 37 °C for 60 min. To evaluate the class of proteolytic enzymes involved, reactions were conducted in parallel in the presence of 10 mM EDTA (a metalloprotease inhibitor) or 10 mM PMSF (a serine protease inhibitor). Fibrinogen alone was used as a negative control. The enzymatic reactions were terminated by the addition of 3× Laemmli sample buffer containing 5% β-mercaptoethanol, followed by heating at 100 °C for 5 min. Samples were resolved by 10% SDS-PAGE under reducing conditions [42]. Gels were stained with PlusOne Coomassie PhastGel™ Blue R-350 (GE Healthcare, Uppsala, Sweden). Degradation of fibrinogen was assessed by analyzing the disappearance or intensity reduction in the characteristic α (66 kDa), β (52 kDa), and γ (46.5 kDa) chains, in comparison to control lanes containing untreated fibrinogen. Molecular weight markers (GE Healthcare, Uppsala, Sweden, 17-0446-01; 14–96 kDa) were used to confirm band identification.

#### 5.5.4. L-Amino Acid Oxidase (LAAO) Activity

LAAO activity was assessed using a microplate-based colorimetric assay following established methodology with adaptations for 96-well microplates [48,49], which detects hydrogen peroxide (H_2_O_2_) production resulting from oxidative deamination of L-leucine. Briefly, scorpion venoms (100 µg/well for BamazV, TmetuV, and TserrV, and 78 µg/well for TsilvV) were incubated with 5 mM L-leucine in 50 mM Tris-HCl buffer (pH 7.0) in a final volume of 200 µL at 37 °C for 60 min. A lower amount of TsilvV (78 µg/well) was used because of limited venom availability, with activity values normalized accordingly. CdtV (50 µg/well) was included as a positive control, given its established LAAO activity [49]. Reactions were stopped by adding 50 µL of 2 M H_2_SO_4_ to each well.

Hydrogen peroxide generation was detected colorimetrically at 492 nm using 2 mM o-phenylenediamine (OPD) (Sigma-Aldrich Co., St. Louis, MO, USA), in the presence of 1 U/mL horseradish peroxidase (HRP, Sigma-Aldrich Co., St. Louis, MO, USA) as chromogenic indicators. Absorbance was read with a reference wavelength of 630 nm using a microplate reader. All assays were performed in triplicate, and blank reactions (substrate without enzyme) were included to correct for background. LAAO activity was expressed as relative activity (%), calculated based on the absorbance at 492/630 nm, with the activity of the positive control (CdtV) set as 100%.

#### 5.5.5. Phosphodiesterase (PDE) Activity

PDE activity was assessed using a colorimetric microplate-based method following the original protocol by Bjork, with microplate adaptations [50,51]. The reaction is based on the enzymatic hydrolysis of bis-p-nitrophenyl phosphate (bis-pNPP), a chromogenic substrate that releases p-nitrophenol, detectable at 400 nm. Briefly, 100 µL of substrate solution (1 mM bis-pNPP in 100 mM Tris-HCl, pH 8.8) was dispensed into each well of a 96-well flat-bottom microplate and pre-incubated at 37 °C for 10 min. Next, scorpion venoms (50 µg/well) or *Crotalus durissus collilineatus* venom (3 µg/well, used as a positive control [36]) were added in a final reaction volume of 150 µL. Blank controls (buffer plus substrate without venom) were included to correct for non-enzymatic hydrolysis. The mixtures were incubated at 37 °C for 30 min, and reactions were terminated by the addition of 50 µL of 50 mM NaOH to stabilize the chromophore. Absorbance was measured at 400 nm using a microplate reader. All assays were performed in triplicate. PDE activity was expressed as relative activity (%), calculated based on the absorbance at 400 nm, with the activity of the positive control set as 100%.

#### 5.5.6. Statistical Analysis

All experiments were performed in triplicate (n = 3), and results are presented as mean ± standard deviation (SD). Statistical comparisons between experimental groups were conducted using one-way analysis of variance (ANOVA) followed by Dunnett’s post hoc test to evaluate differences relative to control conditions (either untreated venom or positive control, depending on the analysis). Differences were considered statistically significant at *p* < 0.05. All analyses were performed using GraphPad Prism software, version 6.0 (GraphPad Software Inc., San Diego, CA, USA).

### 5.6. N-Terminal Sequencing and In Silico Analysis

The major purified component from each scorpion venom was subjected to N-terminal amino acid sequencing via Edman degradation [52], using an automated PPSQ-33A protein sequenator (Shimadzu Co., Kyoto, Japan). Sequences were obtained through multiple degradation cycles, with quality evaluated based on chromatographic signal intensity. The resulting partial primary structures were analyzed using bioinformatics tools to infer molecular identity and functional classification. Sequence similarity searches were performed using BLASTp against the NCBI non-redundant (nr) protein database (http://blast.ncbi.nlm.nih.gov/Blast.cgi, accessed on 19 June 2025), applying default parameters and manually inspecting alignments for conserved toxin motifs and cysteine frameworks.

Putative N-glycosylation sites were predicted using the NetNGlyc 1.0 server (https://services.healthtech.dtu.dk/services/NetNGlyc-1.0/, accessed on 19 June 2025), which employs a neural network-based approach to identify canonical Asn-Xaa-Ser/Thr motifs; residues with prediction scores ≥ 0.5 were considered potential glycosylation sites. Multiple sequence alignment and secondary structure annotation were performed using MultAlin [53] and ESPript [54], respectively, allowing visualization of conserved residues, cysteine pairing patterns, and structural elements.

### 5.7. Multi-Enzymatic and Limited Digestion (MELD) Protocols

The digestion protocol was the same as previously described [55]. Briefly, 10 µg of each dried venom sample was used for digestion and solubilized in 20 µL of 50 mM ammonium bicarbonate (NH_4_HCO_3_), followed by reduction, alkylation, and digestion steps. Reduction was performed using 2 µL of 30 mM dithiothreitol (DTT), followed by incubation for 40 min at 56 °C with shaking at 650 RPM. Alkylation was then carried out by adding 3 µL of 60 mM iodoacetamide (IAA) and incubating the samples in the dark for 30 min at 25 °C. Due to the excess of IAA, a quenching step was performed by adding 2 µL of 60 mM DTT and incubating for 10 min at RT in the dark. During the digestion, two enzyme-to-protein ratios were used across two samples. The enzyme mix was prepared on ice just before use, using 1 mg/mL stock solutions of trypsin, Glu-C, and chymotrypsin in a volume ratio of 1.00/1.00/1.55, respectively. The high-ratio samples were digested using the undiluted enzyme stock solution, while the low-ratio samples were digested using the same stock solution diluted 1:9 in 25 mM NH_4_HCO_3_ and 5 mM CaCl_2_. Both digestions were carried out simultaneously by adding the same volume of each enzyme mix to two separate 10 µg venom protein solutions that had been previously reduced. For the high-ratio condition, the enzyme-to-protein ratios were 1:85 for trypsin and Glu-C, and 1:55 for chymotrypsin. For the low-ratio condition, the ratios were 1:750 for trypsin and Glu-C, and 1:5000 for chymotrypsin. Each sample was incubated for 2 h at 37 °C with shaking at 650 RPM. The reactions were stopped by adding 10% TFA (*v*/*v*) to reach a final pH of approximately 3. Equal volumes from both digestions (high and low ratios) were pooled together. The combined digest was then lyophilized under vacuum and reconstituted to a concentration of 15 pmol/9 µL in H_2_O/TFA (99.9/0.1, *V*/*V*).

### 5.8. LC-MS/MS Analysis

LC-MS/MS analyses were performed on an Acquity M-Class UPLC system (Waters Corp., Milford, CT, USA) coupled to a Q Exactive Plus mass spectrometer (Thermo Scientific, Waltham, MA, USA), operating in nano-electrospray positive ion mode. The trap column used was a Symmetry C18, 5 µm (180 µm × 20 mm), and the analytical column was a HSS T3 C18, 1.8 µm (75 µm × 250 mm), both from Waters Corp. (Milford, CT, USA). Samples were loaded onto the trap column at a flow rate of 20 µL/min in 98% solvent A for 3 min, then separated on the analytical column at a flow rate of 600 nL/min using the following linear gradient: initial condition 98% A; 5 min: 93% A; 60 min: 70% A; 70 min: 60% A; 73 min: 15% A, maintained for 5 min. The column was then re-equilibrated to initial conditions. Solvent A consisted of 0.1% formic acid in water, and solvent B of 0.1% formic acid in acetonitrile. The total runtime was 100 min. The mass spectrometer operated in Top12 data-dependent acquisition mode. MS1 spectra were acquired with the following parameters: mass range 400–1750 *m*/*z*; resolution of 70,000; AGC target of 3 × 10^6^ or maximum injection time of 50 ms. For MS2 spectra, the parameters were: isolation window of 2.0 m/z; normalized collision energy (NCE) of 25; resolution of 17,500; AGC target of 1 × 10^5^ or maximum injection time of 50 ms. The main tune parameters were as follows: spray voltage of 2.3 kV, capillary temperature of 270 °C, and S-Lens RF level set to 50.0. All mass spectrometry raw data generated in this study are publicly available in the jPOST (Japan ProteOme STandard) repository [56].

### 5.9. Bioinformatics Analysis

The analysis was performed using PEAKS Studio X+, version 10.5 (Bioinformatics Solutions Inc., Waterloo, ON, Canada). De novo sequencing was conducted against a custom database named “Scorpions AND Toxin” (13,859 sequences), created from the UniProt database. The following post-translational modifications (PTMs) were used: carbamidomethylation was set as a fixed modification, while oxidation (M) and deamidation (NQ) were set as variable modifications. The maximum number of allowed miscleavages was set to 3. Mass error tolerances were defined as 5 ppm for precursor ions and 0.015 Da for fragment ions. A false discovery rate (FDR) of 0.1% was applied, with a minimum of one unique significant peptide required per protein. De novo-only identifications were considered when the −10logP score was greater than 20. These parameters were chosen to ensure high confidence in protein identifications and to filter out low-confidence matches. For classification purposes, only the top-ranking proteins were considered. Each identified protein was classified either into toxin families or as a cellular component. The diversity of toxin families was assessed using the following formula:

(1) $$\frac{number\;of\;proteins\;\left(protein\;family\right)}{total\;proteins\;detected\;using\;LC-MS/MS}*100,$$

and the quantification of protein families was performed using spectral counting, calculated as follows:

(2) $$\frac{number\;of\;spectra\;for\;a\;protein}{total\;spectra\;for\;proteins\;detected\;using\;LC-MS/MS}*100.$$

## Data Availability

All mass spectrometry raw data generated in this study are publicly available in the jPOST (Japan ProteOme STandard) repository [56], under the following accession numbers: PXD065964 (ProteomeXchange)/JPST003932 (jPOST) for BamazV; PXD065963/JPST003931 for TmetuV; and PXD065962/JPST003930 for TsilvV. The original contributions presented in this study are included in the article/Supplementary Material. Further inquiries can be directed to the corresponding authors.

## Supplementary Materials

The following supporting information can be downloaded at: https://www.mdpi.com/article/10.3390/toxins17080411/s1. File S1: Figures S1 and S2; Figure S1: Multiple sequence alignments of venom proteins from Amazonian scorpions. (**A**) Multiple sequence alignment of BamazPLA_2_ (Peak B59, Figure 1A) with representative scorpion venom phospholipase A_2_ proteins (PLA_2_s). The red box highlights the Asn-X-Ser/Thr sequence, with the asparagine residue marked with a star (★), indicating the predicted N-glycosylation site as identified by the NetNGlyc 1.0 Server. (**B**) Multiple sequence alignment of Tmetu1 (Peak M1, Figure 1B) with representative members of the α-KTx toxin family. The alignment includes the resolved structure 2JP6 (α-KTx from *Tityus obscurus*), P60211 (Tc32 from *T. obscurus*), and two α-KTx sequences from *T. discrepans* (TdK2 and TdK3). Conserved cysteine residues forming the canonical disulfide bridge pattern (C1–C6) are indicated, with secondary structure elements (β-strands and α-helix) based on 2JP6 shown above the alignment. (**C**) Multiple sequence alignment of TsilvMP_A (Peak V1, Figure 1C) with venom metalloproteases from other Tityus species. TsilvMP_A (bottom row) exhibits high sequence identity and similarity to metalloproteases from *T. trivittatus* (TtrivMP_A), *T. fasciolatus* (TfasMP_A), *T. serrulatus* (TserMP_B), and *T. pachyurus* (TpachMP_A/B), as well as to antarease-like proteases from *Tityus* spp. For all figures (**A**–**C**), conserved residues are shaded in black. Identity (ID), similarity (SIM), and molecular weight (MW) values are presented on the right; nd, not determined; MW, theoretical value calculated using ProtParam; MW^1^, experimentally determined [23]. A solid black line indicates amino acid residues identified by Edman degradation; residues identified by mass spectrometry are marked with a dashed line. The alignments and figures were generated using MultAlin [53] and ESPript [54], respectively. The protein sequence data reported in this paper will appear in the UniProt Knowledgebase under the accession numbers C0HMF5 for BamazPLA_2_, C0HMF6 for Tmetu1, and C0HMF7 for TsilvMP_A; Figure S2: Venn diagram illustrating the number of unique and shared proteins identified across the venoms; File S2: Comprises four .xlsx files containing comparative lists of peptides and proteins identified in each Amazonian scorpion venom. All mass spectrometry raw data generated in this study are publicly available in the jPOST (Japan ProteOme STandard) repository [56], under the following accession numbers: PXD065964 (Pro-teomeXchange) / JPST003932 (jPOST) for BamazV; PXD065963 / JPST003931 for TmetuV; and PXD065962 / JPST003930 for TsilvV.

## Abbreviations

The following abbreviations are used in this manuscript:

ACE	Angiotensin-converting enzyme
BamazV	Brotheas amazonicus venom
CdtV	Crotalus durissus terrificus venom
CTAB	Cetyltrimethylammonium bromide
ECE	Endothelin-converting enzyme
EDTA	Ethylenediaminetetraacetic acid
FPLC	Fast protein liquid chromatography
KTx	Potassium channel toxin
LAAO	L-amino acid oxidase
LC-MS/MS	Liquid chromatography–tandem mass spectrometry
MELD	Multi-Enzymatic Limited Digestion
NaTx	Sodium channel toxin
NOB	4-nitro-3-octanoyloxybenzoic acid
NPC2-like	Niemann–Pick disease type C2-like protein
PAGE	Polyacrylamide gel electrophoresis
PDE	Phosphodiesterase
pI	Isoelectric point
PLA2	Phospholipase A2
PLC	Phospholipase C
PLD	Phospholipase D
PMSF	Phenylmethylsulfonyl fluoride
RyR	Ryanodine receptor
SDS-PAGE	Sodium dodecyl sulfate-polyacrylamide gel electrophoresis
SNARE	Soluble N-ethylmaleimide-sensitive factor Attachment Receptor
TmetuV	Tityus metuendus venom
TRU	Turbidity reducing unit
TserrV	Tityus serrulatus venom
TsilvV	Tityus silvestris venom
