# Supplementary material for: Pioneering Comparative Proteomic and Enzymatic Profiling of Amazonian Scorpion Venoms Enables the Isolation of Their First α-Ktx, Metalloprotease, and Phospholipase A2
Source: Toxins (Basel). 2025 Aug 15;17(8):411. doi: 10.3390/toxins17080411 (PMC12390242; doi:10.3390/toxins17080411)
Supplement: Supplementary file 1 [file toxins-17-00411-s001.zip › Supplementary material_File S1/toxins-3794973-supplementary.pdf]

Supplementary Materials: Pioneering Comparative Proteomic and Enzymatic Profiling of Amazonian Scorpion Venoms Enables the Isolation of Their First α-Ktx, Metalloprotease, and Phospholipase A_2

Karla C. F. Bordon [1,*], Gabrielle C. Santos [1], Jonas G. Martins [2], Gisele A. Wiezel [1], Fernanda G. Amorim [3], Thomas Crasset [3], Damien Redureau [3], Loïc Quinton [3], Rudi E. L. Procópio [4] and Eliane C. Arantes [1,*]

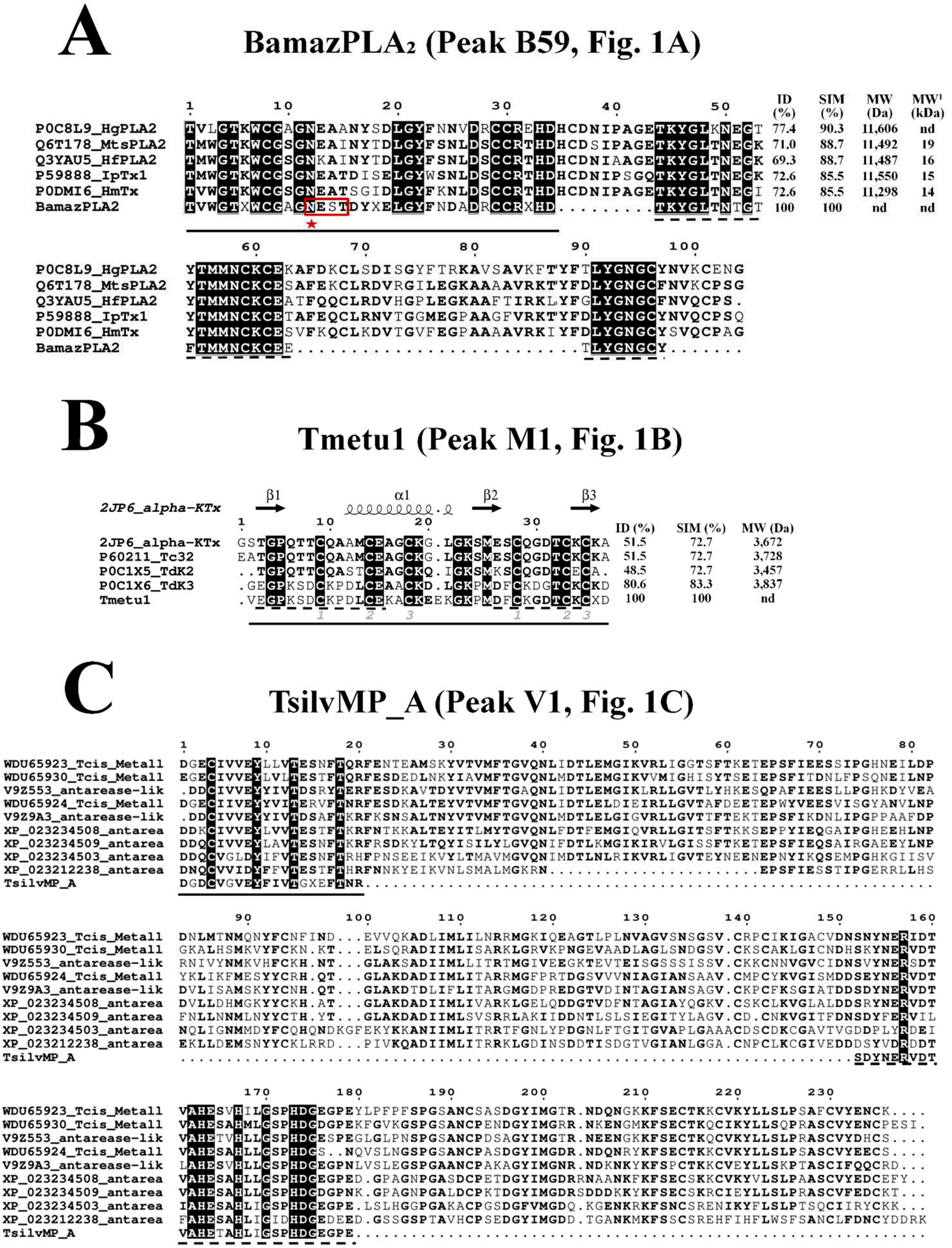
A
BamazPLA2 (Peak B59, Fig. 1A)
1 10 20 30 40 50 ID (%) SIM (%) MW (Da) MW[1] (kDa)
P0C8L9_HgPLA2 TVLGTKWCGAGNEAANYSDLGYFNNVDRCCREHDHCDNIPAGETKYGLKNEGT 77.4 90.3 11,606 nd
Q6T178_MtsPLA2 TMWGTKWCGSGNEAINYTDLGYFSNLDSCCRTHDHCDSIPAGETKYGLTNEGK 71.0 88.7 11,492 19
Q3YAU5_HfPLA2 TMWGTKWCGSGNKAINYTDLGYFSNLDSCCRTHDHCDNIAAGETKYGLTNEGK 69.3 88.7 11,487 16
P59888_IpTx1 TMWGTKWCGSGNEATDISELGYWSNLDSCCRTHDHCDNIPSGQTKYGLTNEGK 72.6 85.5 11,550 15
P0DMI6_HmTx TVWGTKWCGSGNEATSGIDLGYFKNLDSCCRTHDHCDNIPAGETKYGLTNEGI 72.6 85.5 11,298 14
BamazPLA2 TVWGTXWCGAGNESTDYXELGYFNDADRCCRXHD.........TKYGLTNTGT 100 100 nd nd
60 70 80 90 100
P0C8L9_HgPLA2 YTMMNCKCEKAFDKCLSDISGYFTRKAVSAVKFTYFTLYGNGCYNVKCENG
Q6T178_MtsPLA2 YTMMNCKCESAFEKCLRDVRGILEGKAAAAVRKTYFDLYGNGCFNVKCPSG
Q3YAU5_HfPLA2 YTMMNCKCEATFQQCLRDVHGPLEGKAAFTIRKLYFGLYGNGCFNVQCPS.
P59888_IpTx1 YTMMNCKCETAFEQCLRNVTGGMEGPAAGFVRKTYFDLYGNGCYNVQCPSQ
P0DMI6_HmTx YTMMNCKCESVFKQCLKDVTGVFEGPAAAAVRKIYFDLYGNGCYSVQCPAG
BamazPLA2 FTMMNCKCEE.......................TLYGNGCY.......
B
Tmetu1 (Peak M1, Fig. 1B)
2JP6_alpha-KTx β1 α1 β2 β3
1 10 20 30 ID (%) SIM (%) MW (Da)
2JP6_alpha-KTx GSTGPQTTCQAAMCEAGCKG.LGKSMESCQGDTCKCKA 51.5 72.7 3,672
P60211_Tc32 EATGPQTTCQAAMCEAGCKG.LGKSMESCQGDTCKCKA 51.5 72.7 3,728
P0C1X5_TdK2 ..TGPQTTCQASTCEAGCKQ.IGKSMKSCQGDTCECA. 48.5 72.7 3,457
P0C1X6_TdK3 .GEGPKSDCKPDLCEAACKD.LGKPMDFCKDGTCKCKD 80.6 83.3 3,837
Tmetu1 .VEGPKSDCKPDLCEKACKEEKGKPMDFCKGDTCKCXD 100 100 nd
1 2 3 1 2 3
C
TsilvMP_A (Peak V1, Fig. 1C)
1 10 20 30 40 50 60 70 80
WDU65923_Tcis_Metall DGECIVVEYLLVTESNFTQRFENTEAMSKYVTVMFTGVQNLIDTLEMGIKVRLIGGTSFTKETEPSFIEESSIPGHNEILDP
WDU65930_Tcis_Metall EGECIVVEYLVLTESTFTQRFESDEDLNKYIAVMFTGVQNLMDTLEMGIKVVMIGHISYTSEIEPSFITDNLFPSQNEILNP
V9Z553_antarease-lik .DDCIVVEYYIVTDSRYTERFESDKAVTDYVTVMFTGAQNLIDTLEMGIKLRLLGVTLYHKESQPAFIEESLLPGHKDYVEA
WDU65924_Tcis_Metall DGECIVVEYYIVTERVFTNRFESDKALTEYVTVMFTGVQNLIDTLELDIEIRLLGVTAFDEETEPWYVEESVISGYANVLNP
V9Z9A3_antarease-lik .DDCIVVEYYIVTDSAFTKRFKSNSALTNYVTVMFTGVQNLMDTLELGIGVRLLGVTTFTEKTEPSFIKDNLIPGPPAAFDP
XP_023234508_antarea DDKCIVVEYLVVTESTFTKRFNTKKALTEYITLMYTGVQNLFDTFEMGIQVRLLGITSFTKKSEPPYIEQGAIPGHEEHLNP
XP_023234509_antarea DDQCIVVEYLAVTESNFTKRFRSDKYLTQYISILYLGVQNIFDTLKMGIKIRVLGISSFTKETEPSFIEQSAIRGAEEYLNP
XP_023234503_antarea DDQCVGLDYIFVTESNFTRHFPNSEEIKVYLTMAVMGVQNIMDTLNLRIKVRLIGVTEYNEENEPNYIKQSEMPGHKGIISV
XP_023212238_antarea DNQCVVIDYFFVTESTFTHRFNNKYEIKVNLSMALMGKRN.......................EPSFIESSTIPGERRLLHS
TsilvMP_A DGDCVGVEYFIVTGXEFTNR...
90 100 110 120 130 140 150 160
WDU65923_Tcis_Metall DNLMTNMQNYFCNFIND...EVVQKADLIMLILNRRMGKIQEAGTLPLNVAGVSNSGSV.CRPCIKIGACVDNSNYNERIDT
WDU65930_Tcis_Metall GKALHSMKVYFCKN.KT...ELSQRADIIMLISARKLGRVKPNGEVAADLAGLSNDGSV.CKSCAKLGICNDHSKYNERVDT
V9Z553_antarease-lik RNIVYNMKVHFCKH.NT...GLAKSADIIMLLITRTMGIVEEGKTEVTEISGSSSISSV.CKKCNNVGVCIDNSVYNERSDT
WDU65924_Tcis_Metall YKLIKFMESYYCRH.QT...GLAKDADIIMLITARRMGFPRTDGSVVVNIAGIANSAAV.CMPCYKVGISMDDSEYNERVDT
V9Z9A3_antarease-lik DVLISAMSKYYCNH.QT...GLAKDTDLIFLITARGMGDPREDGTVDINTAGIANSAGV.CKPCFKSGIATDDSDYNERVDT
XP_023234508_antarea DVLLDHMGKYYCKH.AT...GLAKDADIIMLIVARKLGELQDDGTVDFNTAGIAYQGKV.CSKCLKVGLALDDSRYNERVDT
XP_023234509_antarea FNLLNNMLNYYCTH.YT...GLAKDADIIMLSVSRRLAKIIDDNTLSLSIEGITYLAGV.CD.CNKVGITFDNSDYFERVIL
XP_023234503_antarea NQLIGNMMDYFCQHQNDKGFEKYKKANIIMLITRRTFGNLYPDGNLFTGITGVAPLGAAACDSCDKCGAVTVGDDPLYRDEI
XP_023212238_antarea EKLLDEMSNYYCKLRRD...PIVKQADIIMLITRRKLGDINSDDTISDGTVGIANLGGA.CNPCLKCGIVEDDDSYVDRDDT
TsilvMP_A ....SDYNERVDT
170 180 190 200 210 220 230
WDU65923_Tcis_Metall VAHESVHILGSPHDGEGPEYLPFPFSPGSANCSASDGYIMGTR.NDQNGKKFSECTKKCVKYLLSLPSAFCVYENCK....
WDU65930_Tcis_Metall VAHESAHMLGSPHDGDGPEKFGVKGSPGSANCPENDGYIMGRR.NKENGMKFSECTKQCIKYLLSQPRASCVYENCPESI.
V9Z553_antarease-lik VAHETVHLLGSPHDGESPEGLGLPNSPGSANCPDSADGYIMGTR.NEENGKKFSECTKQCVKYLLSLPRASCVYDHCS....
WDU65924_Tcis_Metall VAHESAHLLGSPHDGS..NQVSLNGSPGSANCPASDGYIMGDR.NDQNRYKFSECTKKCVKYLLSLPSASCVYEECS....
V9Z9A3_antarease-lik LAHESVHLLGSPHDGEGPNLVSLEGSPGAANCPAKAGYIMGNR.NDKNKYKFSPCTKKCVEYLLSKPTASCIFQQCRD...
XP_023234508_antarea VAHESAHLLGSPHDGEGPED.GPAGNPGASDCPETDGYIMGDRRNAANKFKFSECSKKCVKYVLSLPAASCVYEDCEFY..
XP_023234509_antarea VAHESAHLLGSPHDGDGPNK.GPAGNPGALDCPKTDGYIMGDRSDDDKKYKFSECSKRCIEYLLSLPRASCVFEDCKT...
XP_023234503_antarea IAHESAHLIGSPHDGEGPE.LSLHGGPGAKACPGSDGFVMGDQ.KGENKRKFSNCSRENIKYFLSLPTSQCIIRYCKK...
XP_023212238_antarea FAHESAHLIGIDHDGEDEED.GSSGSPTAVHCPSEDGYIMGDD.TGANKMKFSSCSREHFIHFLWSFSANCLFDNCYDDRK
TsilvMP_A VAHETAHLIGSPHDGEGPE..

Figure S1. Multiple sequence alignments of venom proteins from Amazonian scorpions. (**A**) Multiple sequence alignment of BamazPLA_2 (Peak B59, Fig. 1A) with representative scorpion venom phospholipase A_2 proteins (PLA_2s). The red box highlights the Asn-X-Ser/Thr sequence, with the asparagine residue marked with a star (★), indicating the predicted N-glycosylation site as identified by the NetNGlyc 1.0 Server. (**B**) Multiple sequence alignment of Tmetu1 (Peak M1, Fig. 1B) with representative members of the α-KTx toxin family. The alignment includes the resolved structure 2JP6 (α-KTx from *Tityus obscurus*), P60211 (Tc32 from *T. obscurus*), and two α-KTx sequences from *T. discrepans* (TdK2 and TdK3). Conserved cysteine residues forming the canonical disulfide bridge pattern (C1–C6) are indicated, with secondary structure elements (β-strands and α-helix) based on 2JP6 shown above the alignment. (**C**) Multiple sequence alignment of TsilvMP_A (Peak V1, Fig. 1C) with venom metalloproteases from other *Tityus* species. TsilvMP_A (bottom row) exhibits high sequence identity and similarity to metalloproteases from *T. trivittatus* (TtrivMP_A), *T. fasciolatus* (TfasMP_A), *T. serrulatus* (TserMP_B), and *T. pachyurus* (TpachMP_A/B), as well as to antarease-like proteases from *Tityus* spp. For all figures (**A-C**), conserved residues are shaded in black. Identity (ID), similarity (SIM), and molecular weight (MW) values are presented on the right; nd, not determined; MW, theoretical value calculated using ProtParam; MW^1, experimentally determined (Krayem and Gargouri, 2020). A solid black line indicates amino acid residues identified by Edman degradation; residues identified by mass spectrometry are marked with a dashed line. The alignments and figures were generated using MultAlin (Corpet, 1988) and ESPript (Robert and Gouet, 2014), respectively. The protein sequence data reported in this paper will appear in the UniProt Knowledgebase under the accession numbers C0HMF5 for BamazPLA_2, C0HMF6 for Tmetu1, and C0HMF7 for TsilvMP_A.

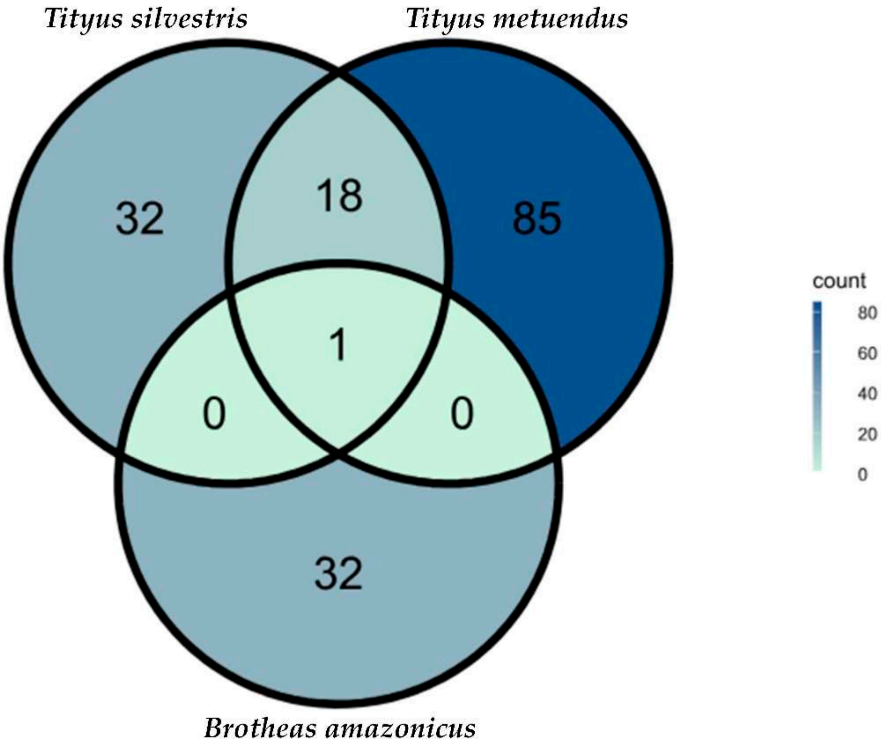

Figure S2. Venn diagram illustrating the number of unique and shared proteins identified across Amazonian scorpion venoms. File S2 comprises four Microsoft Excel (.xlsx) files detailing comparative peptide and protein identifications for each venom.
