# Supplementary figures and images for: Pioneering Comparative Proteomic and Enzymatic Profiling of Amazonian Scorpion Venoms Enables the Isolation of Their First α-Ktx, Metalloprotease, and Phospholipase A2
Source: Toxins (Basel). 2025 Aug 15;17(8):411. doi: 10.3390/toxins17080411 (PMC12390242; doi:10.3390/toxins17080411)

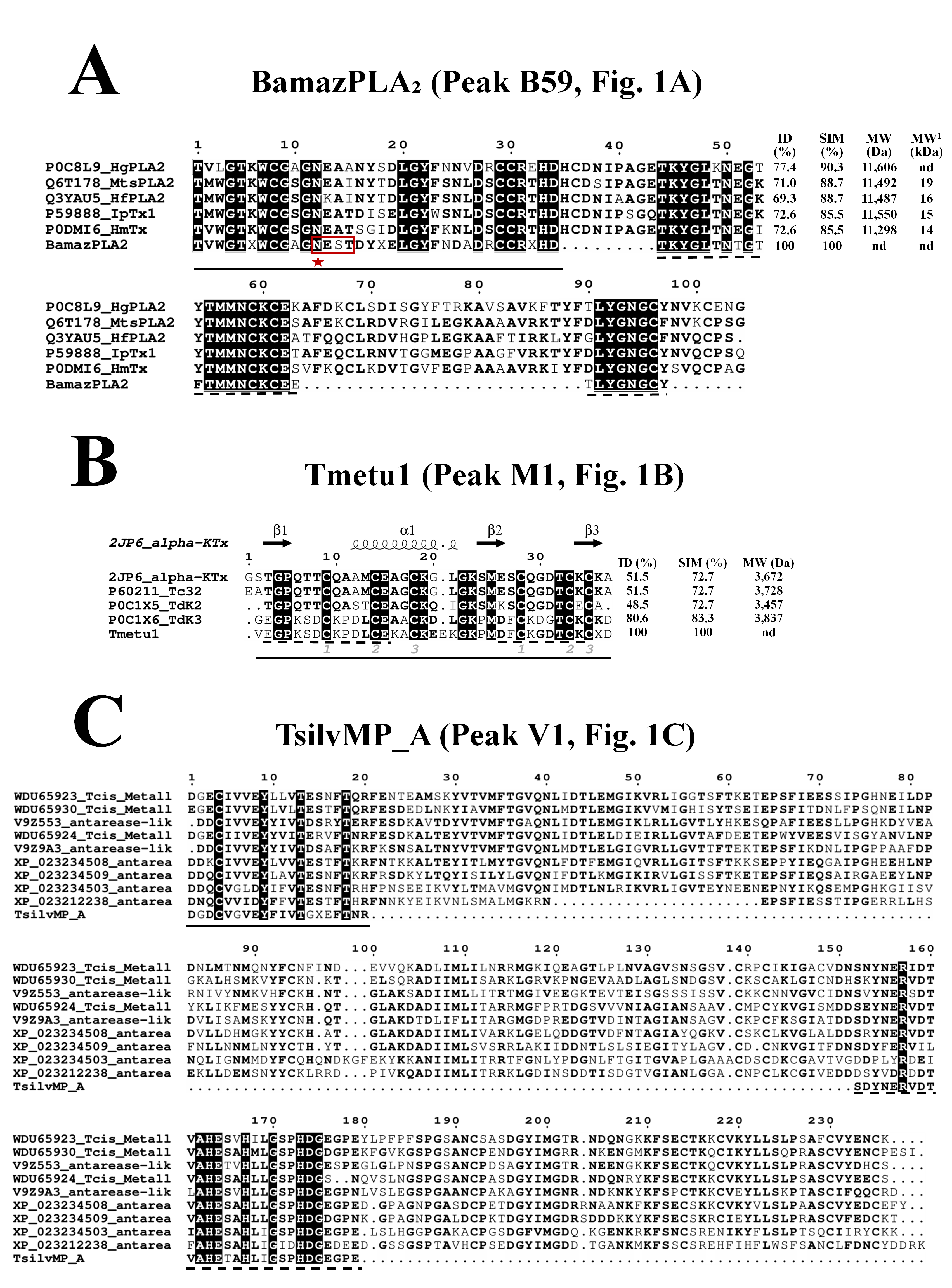

Supplement: Supplementary file 1 [file toxins-17-00411-s001.zip › Supplementary material_File S1/Figure S1.tif]

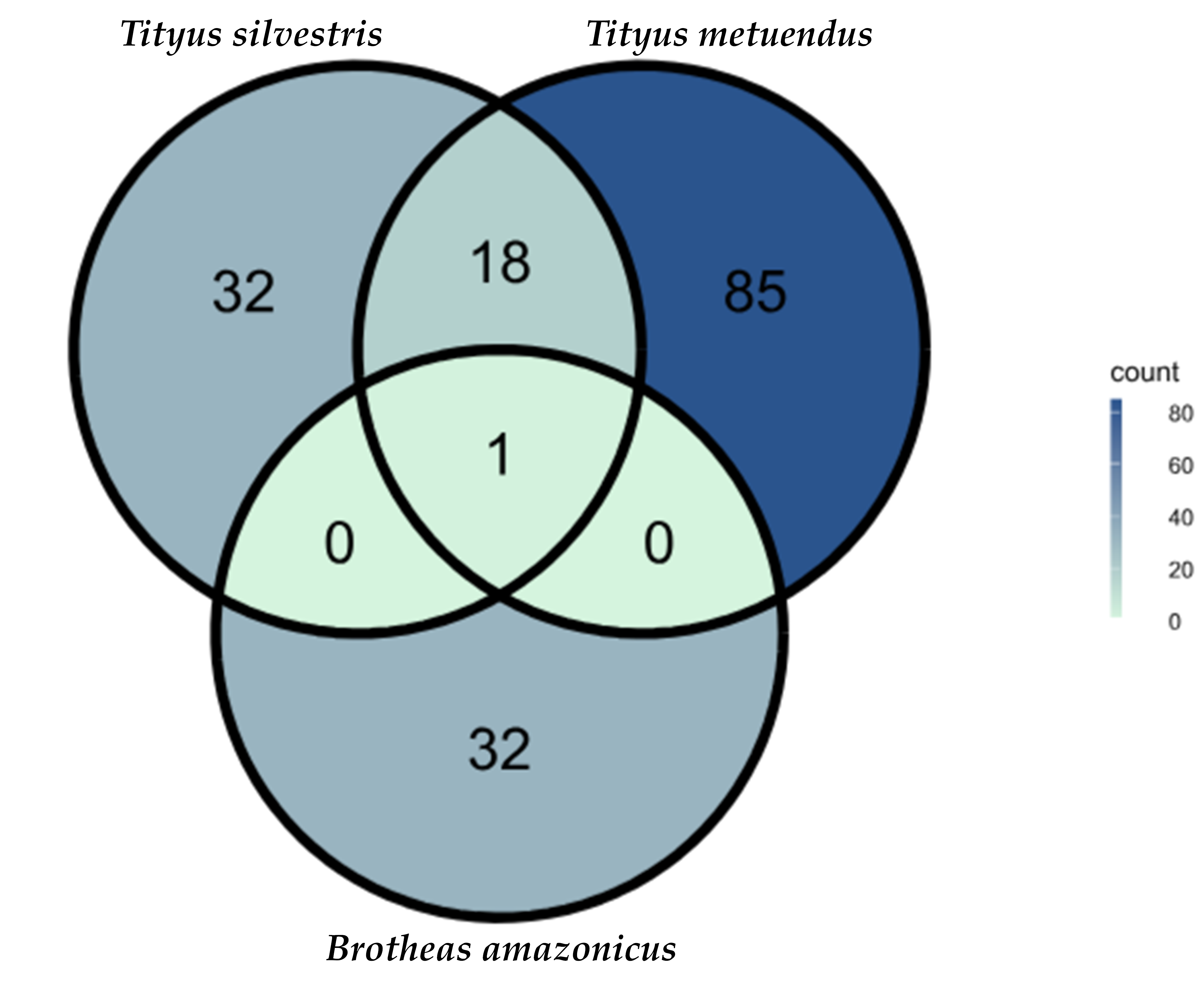

Supplement: Supplementary file 1 [file toxins-17-00411-s001.zip › Supplementary material_File S1/Figure S2.tif]
